# 

**Published:** 1886-04

**Authors:** 


*To State and Local Dental Societies :*

Remember that every local society which has adopted substantially the code of ethics of the American Dental Association, is entitled to one delegate for every five members. Appoint your delegates soon and let all unite in making this the largest and most profitable meeting of the Association ever yet held. J. N. CROUSE,

*Chairman of Executive Committee.*

CHICAGO, April 16, 1886.

*To the Members of the American Dental Association :*

The votes of nearly all the members have been received. A majority of the votes cast are in favor of Chicago over all other places. And a very large majority pledge their attendance if the meeting shall be held in Chicago. But in deference to the minority and for the sake of harmonizing all differences, as Chairman of the Executive Committee and Committee of Arrangements, I hereby, with the consent of my Colleagues, announce the next place of meeting to be at Niagara Falls, August 3.

Information concerning Hotel and Railroad rates will be given later.

J. N. CROUSE,
2101 Michigan Ave. *Chairman of Executive Committee.*

---

### Editorial, Etc.

### To Readers and Correspondents.

Communications intended for publication, and exchange journals and papers should be addressed to the Editor of the American Journal of Dental Science, 259 N. Eutaw St., Baltimore, Md. All letters relating to the business of the Journal, or containing cash remittances, to be directed to Messrs. Snowden & Cowman. Articles for publication should be received by the editor at least three weeks previous to the first month on which the number in which it is designed for them to appear, is issued.

☞ The American Journal of Dental Science will contain fifty pages of reading matter in each number, making a volume of about *Six Hundred* pages,

EXCLUSIVE OF ADVERTISEMENTS.

